# Clinical Characteristics and Genetic Etiology of Children With Developmental Language Disorder

**DOI:** 10.3389/fped.2021.651995

**Published:** 2021-07-01

**Authors:** Marielle B. Plug, Vivian van Wijngaarden, Hester de Wilde, Ellen van Binsbergen, Inge Stegeman, Marie-José H. van den Boogaard, Adriana L. Smit

**Affiliations:** ^1^Department of Otorhinolaryngology Head and Neck Surgery, University Medical Center Utrecht, Utrecht, Netherlands; ^2^Department of Genetics, University Medical Center Utrecht, Utrecht, Netherlands; ^3^Brain Center, University Medical Center Utrecht, Utrecht, Netherlands; ^4^Epidemiology and Data Science, Amsterdam University Medical Centers, University of Amsterdam, Amsterdam, Netherlands

**Keywords:** children, genes, developmental language disorder (DLD), diagnostic, genetic etiology

## Abstract

**Objective:** Developmental language delay (DLD) is one of the most common disabilities in childhood and can negatively affect a child's communication skills and academic and/or psychosocial development. To date, an increasing number of causative genes have been identified by diagnostic techniques like next generation sequencing. An early genetic diagnosis is important to properly prepare and counsel children and parents for possible future difficulties. Despite this, genetic assessment is usually not part of a standardized diagnostic set in children with developmental language delay. In this study, we aim to assess the diagnostic outcomes of children primarily assessed for speech and language delay who were subsequently referred for genetic etiological assessment.

**Methods:** Medical records of children referred to the department of Otorhinolaryngology of the Wilhelmina Children's Hospital for diagnostic work-up for a suspected speech and language delay between June 2011 and December 2018 who were additionally referred to a geneticist were evaluated. Study parameters concerning medical history, behavioral problems, language development, intelligence, and hearing were recorded. Outcomes of genetic analysis were evaluated.

**Results:** A total of 127 patients were diagnosed with a developmental language delay. Genetic analysis was conducted in 119 out of 127 patients with a language delay and eligible for this study. The median time between initial speech and language assessment and the first genetic consultation was 10 months (IQR 5.0–23.0). In 34 out of 127 patients a causative genetic diagnosis was found to explain their DLD.

**Conclusion:** In approximately a quarter of the patients (26.8%) diagnosed with developmental language delay, a causative genetic diagnosis was confirmed. This demonstrates the opportunity to identify an underlying genetic etiology in children with developmental language delay. However, in order to optimize the diagnostic process and clinical care for these children, two important research gaps need to be addressed. First, research should focus on assessing the clinical impact and effect on treatment outcomes of a genetic diagnosis. Secondly, it is important to recognize for which children genetic testing is most beneficial.

## Introduction

Developmental language delay (DLD) is one of the most common disabilities in childhood (1). The term is used to refer to problems in one or more components of language (i.e., phonology, morphosyntax, semantics or pragmatics). Often, a distinction is made between a receptive and/or an expressive language disorder. Receptive language refers to the ability to understand the meaning of words and sentences. Expressive language refers to the ability to put thoughts and feelings into words and sentences, in a grammatically, and semantically accurate manner (2). The reported prevalence of children with atypical speech and language development varies with ranges reported from 2.0 to 7.0%, depending on study population and used definitions (3–6). If this delay remains untreated, it can negatively affect a child's communication skills and academic and/or psychosocial development (7). For example, children with a speech or language delay are twice as likely to develop emotional problems, and behavioral difficulties compared to typically developing peers (8). Reported risk factors for speech and language delays are diverse, though low parental education, a family history of developmental communication disorder, male sex, behavioral disorder, and low birth weight are the most consistently reported (9–11). Furthermore, language disorders in children receive less recognition and less research funding than other neurodevelopmental conditions, such as autism. Despite the importance of a developmental language disorder for the daily life of those affected, little scientific research has been performed (12).

In case of a primary language delay, also referred to as DLD or specific language impairment, no specific cause like a atypical sensory development or insufficient language exposure can be found (2). In secondary language delays, conditions, such as autism, epilepsy, hearing loss or general developmental disabilities are considered to be the origin (13). Therefore, in children not meeting the expected developmental language milestones, an extensive multidisciplinary assessment is essential.

Several studies have documented that speech and language deficiencies can be heritable and certain causative genes, such as ATP2C2, FOXP2, and CMIP have been identified (14–16). Furthermore, some genetic syndromes are known to be associated with a delayed language development, like 22q11 deletion syndrome, and sex chromosomal aneuploidy (17, 18). Recent technological advances in DNA sequencing and gene discovery have led to an explosive growth of knowledge about potential genetic components in communication disorders. This provides opportunities to identify an underlying genetic etiology and could end the parent's/child's quest for a cause for the delay.

However, at this moment, genetic assessment is usually not part of a standardized diagnostic work-up for children with a delayed language development. Considering the technological advancements made in genetic analysis, with lowering costs of genetic testing and shorter turnaround times, genetic analysis could be offered during the primary work-up for children presenting with speech, and language delays (19). However, before implementing this in daily practice, more information is needed about the present-day diagnostic outcome of children with delayed language development. In this retrospective study we aim to assess the diagnostic/genetic outcomes and time-delay of children diagnosed with a DLD who were subsequently referred for genetic etiological assessment.

## Methods

### Study Design and Setting

In this single center retrospective cohort study, data was collected from children who were diagnosed with DLD after diagnostic work-up by a specialized team of the department of Otorhinolaryngology of the Wilhelmina Children's Hospital/University Medical Center Utrecht (UMCU) in The Netherlands. Ethical approval for this study was obtained from the UMCU ethical committee (local number: 19-481/C). A waiver of consent was granted by the retrospective nature of the data collection.

### Participants

Children were included if they were diagnosed with DLD by the Speech and Language team of the department of Otorhinolaryngology of the Wilhelmina Children's Hospital Utrecht in the Netherlands after diagnostic work-up for a suspected language delay. A cohort of children were selected when consultation took place between June 2011 and December 2018. The diagnostic work-up consisted of a single-day multidisciplinary assessment including an evaluation by an otorhinolaryngologist, an audiologist, a speech-language pathologist, and a behavioral therapist. In several patients, multiple visits were planned within several weeks after the first consultation for additional testing [e.g., intelligence level test, such as Schlichting and Clinical Evaluation of Language Fundamentals, Version IV (CELF-IV) test]. Only patients with a language delay who received a genetic assessment by the department of Genetics of the Wilhelmina Children's Hospital following this first multidisciplinary consultation were finally included into the study. Data from these consultations and specialized testing was extracted from the electronic medical files.

### Study Parameters

The medical records were retrospectively analyzed by only one researcher (MP) to score demographic, medical, hearing, speech- and language, behavior, and developmental characteristics as recorded during the multidisciplinary assessment. Demographic characteristics included sex, age at presentation, consanguinity of parents (relationship by descent from a common ancestor, e.g., cousin and niece), adoption status, specialty of referrer, and initial reason for referral for speech and language assessment and genetic testing.

### Medical History Assessment

The medical history was assessed by an otorhinolaryngologist with the parents/caretakers of the child. The following parameters were obtained: pregnancy characteristics (i); maternal or neonatal difficulties during pregnancy (e.g., cardiovascular, pregnancy/child birth (e.g., twin birth, breech birth) as classified according to the International Classification of Diseases (ICD) 11th revision, June 2018 (20)). Delivery characteristics (ii) included gestational age (defined as the time between the first day of the last menstrual period and the day of delivery in weeks) (21), delivery mode and birth weight. Neonatal outcome (iii): presence at a neonatal emergency, observed malformations of the head/neck in neonatal period. Medical history/co-morbidities: history of tympanostomy tubes, adenectomy, tonsillectomy or clipped tongue strap, other surgical procedures. Presence of epilepsy or abnormal vision (based on information of childcare center/parents). Hearing characteristics (iv): outcome of neonatal hearing screening, family history of hearing loss (first and second degree). Speech/language, breathing, and ingestion characteristics (v): excessive saliva loss, breath stops during sleep (apnoea), difficulties with swallowing solid food, nasal speech, problems with sucking/drinking/food, aberrant mouth behavior (e.g., drooling, open mouth behavior). A positive family history of speech and language delay was documented when family members in the first and second degree (e.g., parents, children, siblings) presented with this symptom.

### Physical Examination

Physical examination included morphological anomalies of the head/neck, abnormalities at otoscopic evaluation (e.g., otitis media with effusion, tympanostomy tube, ear wax), rhinoscopic observations (e.g., rhinitis), fiber optic evaluation of the naso-, and oropharynx (e.g., adenoid obstruction, velar hypotonia) if performed. Abnormalities of the jaw (e.g., retrognathia), palate (e.g., palatal cleft or bifid uvula), mouth (e.g., enlarged tonsils), and voice or speech (hyponasal or hypernasal speech) were also recorded.

### Hearing Assessment

The child's hearing level was assessed by the audiologist with tests depending on age and set limits for sufficient hearing to develop a normal speech and language (for at least one ear) as described in Appendix 4 (22, 23).

### Speech and Language Assessment

To assess the development of speech and language, the language production of the child during consultation was observed by a speech language pathologist and related to Dutch national speaking standards (24). Additional tests for language perception and language production were performed during a second consultation by a speech therapist, with tests depending on age (25, 26). An overview of the performed language tests per age is provided in Appendix 4. In this study, a language delay was diagnosed when it resulted in a functional impairment in everyday life, unlikely to resolve without the help of a speech therapist (12). When the language delay was not associated with a known biomedical etiology (e.g., brain injury, hearing loss, autism), the term developmental language disorder (DLD) was used. (12).

### Developmental and Behavior Assessment

General development of the child was assessed by the behavioral therapist. The following data were retrieved from medical records: developmental characteristics; first steps (reported in months), motor development compared to peers (reported as normal or delayed based on parent's information), and history of physical therapy. In order to interpret the developmental level, motor development was considered to be delayed when the child started walking after the age of 18 months. Besides this, behavioral prominences (e.g., reaction to verbal stimuli/response to assignments) during consultation were observed by the behavioral therapist, and were scored as normal or abnormal. Motor skills were scored with validated questionnaires and the Intelligence Quotient (IQ) was tested (during a second consultation) using tests depending on age/based on the demand of the applicant, as described in Appendix 4 (27–29). Additionally, the significance between the verbal and performance intelligence (e.g., harmonic/disharmonic profile) was calculated by methods described in the guidelines of the used test (30–32). To gain insight into the level of education of the parents, the level of education was categorized into the following categories (33): (i) primary education (lower educational level) contained primary school and the first phase of secondary education (Lower General Secondary Education and the first 3 years of Higher General Secondary Education/Pre-university education). (ii) secondary education contained education for which admission requirements apply and for which primary education has been completed (Intermediate vocational education, last years of Higher General Secondary Education/Pre-university education). (iii) higher level of education consisted of education with scientific or vocational knowledge (Higher vocational education, University, PhD) (34).

### Genetic Testing

Genetic testing was performed in those children referred to the geneticist of our center by the speech and language team or referred by other medical specialties (e.g., pediatrician/neurologist) for multiple reasons (e.g., developmental delay with language delay as a symptom). The genetic tests performed included: Single Nucleotide Polymorphism (SNP) array, Karyotyping analysis, repeat expansion testing (for fragile X-syndrome), specific gene panel analysis based on current knowledge of genes causing specific abnormalities in the child (e.g., deafness, language delay, developmental delay) and (trio) Whole Exome Sequencing (WES). Resulting variants were assessed by a laboratory specialist and classified on pathogenicity according to the International Standards for Cytogenomic Arrays (ISCA) and American College of Medical Genetics (ACMG) guidelines (e.g., type 1–5) (35, 36). These results were re-evaluated for causality by a geneticist based on current knowledge. Genetic findings were divided into three causative subcategories: “possibly pathogenic,” “probably pathogenic,” and “certainly pathogenic,” based on whether the variant, or gene(s) within the variant, was known to be disease-associated in databases like OMIM and Human Gene Mutation Database (HGMD) (37, 38). PubMed was also used to determine whether a variant or gene had been previously described as a cause for developmental language problems (39). A “possible cause” was defined as a new (*de novo*) variant containing or affecting a likely causing gene. A “probable cause” was defined as a variant containing or affecting a known gene where pathogenicity of the variant is not certain (variant of unknown significance: VUS). A “certain cause” was defined as a variant classified as pathogenic (5P), in a gene previously associated with language or developmental disorders with language delay as a symptom. Time-delay was defined as the time between initial multidisciplinary speech and language assessment and time of first genetic consultation (to initiate genetic testing), and time of genetic diagnosis.

### Data Extraction

The data from the medical records of the included patients were pseudonymised. Variables and outcomes were extracted from the clinical reports by a single researcher. In case of doubt about the interpretation of the notes, the expert of the MD team or geneticist was contacted to reach consensus about the interpretation. Due to the nature of the study, the outcome measures were not blinded from the outcome assessors.

### Statistical Analysis

Descriptive statistics were used to provide an overview of the study population. Differences in characteristics between patients diagnosed with a developmental language disorder were calculated by using Chi-square test or Fisher's exact test. Baseline characteristics were compared between patients with DLD with a genetic etiological diagnosis vs. patients with DLD without by using Chi-square test. Genetic outcomes per genetic test were provided using descriptive statistics. *P*-values below 0.05 were considered as statistically significant. IBM SPSS statistics 25 (SPSS Inc., Chicago, IL, USA) was used for all analyses.

## Results

### Description of Cohort

A total of 127 patients were eligible for this study. Of all 127 patients, 77 (60.6%) were male (Table 1). Mean age at initial presentation was 3.7 years old (SD 3.7). The majority of children were referred to the speech and language team for the diagnostic work-up by a general practitioner (*n* = 61; 50.8%). The most common reasons for referral were a delay in speech and language development (*n* = 69; 56.2%) and no progress in speech, and language development despite speech therapy (*n* = 53; 43.1%).

**Table 1:** Overview of patients diagnosed with developmental language disorder (DLD) who were additionally assessed by a geneticist.

	**Total cohort *N* = 127, (%)**
Sex, *n*	
Male	77 (60.6)
Female	50 (39.4)
Age years, (SD)	3.7 (3.7)
Referred by	
General practitioner	6/120 (50.8)
Speech therapist	7/120 (5.8)
Physician	8/120 (6.7)
Pediatrician	23/120 (19.2)
Reason for referral	
Delayed speech/language development	69/123 (54.3)
Insufficient effect of speech therapy	53/123 (41.7)
Regression or decline speech/language	1/123 (0.8)
Duration of pregnancy (SD), wks	38.9 (2.23)
Birth weight (IQR) (*n* = 109), g (mean, range)	3.231 (670–5.500)
<1.500	2 (1.8)
1.500–2.500	12 (12.0)
>2.500	93 (85.3)
Difficulties during pregnancy	37/124 (29.1)
Perinatal emergency	11/127 (8.7)
Co-morbidities	22/127 (17.3)
Medical history	
TT	30/126 (23.8)
Adenectomy	26/125 (20.8)
Tonsillectomy	13/125 (10.4)
Clipped tongue strap	4/116 (3.5)
Facial dysmorphology head/neck area^c^	10/117 (8.5)
Motor delay^a^	38/117 (29.9)
Observed abnormalities in behavior	59/116 (51.0)
Maternal educational level	
Primary education	27/124 (21.8)
Secondary education	47/124 (37.9)
Higher to tertiary education	50/124 (0.3)
Excessive saliva loss	34/126 (27.0)
Presence of breathing stops^d^	6/123 (4.9)
Difficulties swallowing solid food	12/123 (9.8)
Nasal speech	11/123 (8.9)
Insufficient vision	18/115 (15.7)
Consanguinity	8/121 (6.6)
FH of speech and language delay^b^	72/107 (67.3)
FH of hearing impairment in childhood^b^	29/124 (23.4)

*n, number; SD, standard deviation around the mean; IQR, interquartile range around the median; m, months; wks, weeks, g, gram, TT, tympanostomy tubes; FH, family history*.

a *Motor delay = walking >18 months*.

b *First and second degree*.

c *Directly after birth*.

d *Based on parent's information*.

Co-morbidities occurred in 22 (17.3%) of the patients and motor delay was present in 38 (32.5%) of the children. Specifications of the type of co-morbidities are noted in Appendix 1.

The behavioral specialist subjectively observed abnormalities in behavior in 59 (51%) of cases during the initial assessment (e.g., abnormal eye contact, limb movements). Dysmorphic features of the head/neck area were seen in 10 patients (8.5%) (e.g., plagiocephaly, hypertelorism, wide nose bridge). Maternal difficulties during pregnancy (e.g., pre-eclampsia, gestational diabetes, hypertension) occurred in 37 cases (29.8%), of which cardiovascular difficulties (mostly hypertension) were the most common (*n* = 11, 29.7%). Specifications of maternal difficulties and perinatal emergency are listed in Appendix 2. Additional baseline demographic characteristics of the study cohort are listed in Table 1.

### Medical, Hearing Examination and Developmental Outcomes

Otoscopy was normal in the majority (left ear vs. right ear) (*n* = 80, 63% vs. *n* = 81, 63.8%) (Table 2). Otoscopy was abnormal (e.g., Otitis Media, Otorrhoea) (left ear vs. right ear) 47 vs. 17 patients (37, 36.2%). Palatal abnormalities included cleft palate (*n* = 1, 12.5%), bifid uvula and a gothic palate (*n* = 6.75%). Hearing level was mostly normal (*n* = 99, 78.0%). Outcomes of additional developmental tests performed after initial assessment in children diagnosed with DLD are provided in Table 2. Intelligence tests were performed in 60 out of 127 (47.3%) patients, the majority (*n* = 39, 65%) had an IQ score below 75.

**Table 2:** Otologic examinations and additional developmental tests in children with developmental language disorder (DLD).

	**Total cohort *N* = 127, (%)**
Abnormalities head/neck area^a^	34/120 (28.3)
Otoscopy left ear	
Normal	80/127 (63.0)
Abnormal	47/127 (37.0)
Otitis media (OMA/OME)	31/47 (66.0)
Not classified^b^	5/47 (10.6)
Otorrhoea	3/47 (6.4)
Otoscopy right ear	
Normal	81/127 (63.8)
Abnormal	46/127 (36.2)
Otitis media (OMA/OME)	26/44 (59.1)
Not classified^b^	4/44 (9.1)
Otorrhoea	2/44 (4.6)
Rhinoscopy	
Abnormal	31/125 (24.8)
Teeth + jaw	
Abnormal	8/124 (6.5)
Palate	
Abnormal	8/125 (6.4)
Tonsils	
Abnormal^c^	17/122 (13.9)
After tonsillectomy	10/122 (8.2)
Voice and speech	
Abnormal	23/108 (21.2)
Hyponasal	7/23 (30.4)
Hypernasal	4/23 (17.4)
Hearing level	
Sufficient	99/127 (78.0)
Insufficient	19/127 (15.0)
IQ (tested)	*N* = 60
Intellectual disability^d^	39 (65)
Below average level	19 (31.7)
BSID (tested)	*N* = 30
Below average level	26 (86.7)
Average level	4 (13.3)

*SD, standard deviation around mean; OME, otitis media with effusion; OMA, otitis media acuta; TT, tympanostomy tube*.

a *Observed dysmorphic features in head/neck area observed during life*.

b *Tympanic membrane not inspected due to ear wax or resistance of the child*.

c *Abnormal defined as enlarged tonsils*.

d *IQ below 75*.

### Genetic Outcomes

Of all 127 patients included in this study, 75 (59.1%) were initially referred by a pediatrician to a clinical geneticist for genetic analysis. The most common primary reason for referral to the geneticist that was noted in the medical records of the studied children was a developmental disorder (*n* = 44, 34.6%). Specifications of the referrer and reason of referral to the geneticist can be found in Appendix 3. The median time between the speech and language assessment and the first genetic consultation and genetic diagnosis was 10.0 months (IQR 5.0–23.0), and 12.0 months (IQR 6.0–25.0) respectively.

**Figure 1:**
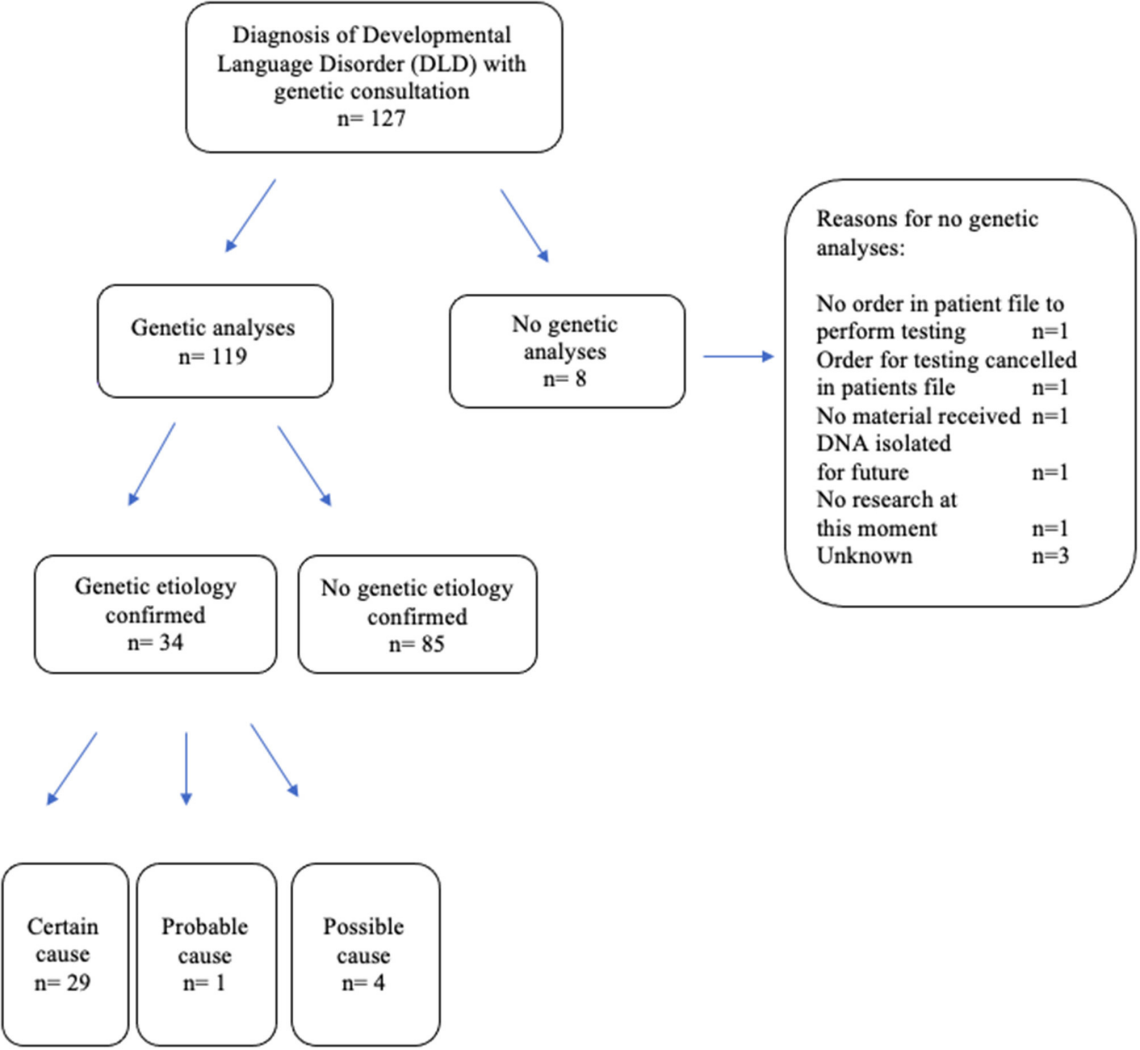
Included study patients with developmental language disorder (DLD).

Genetic testing was conducted in 119 out of 127 children (93.7%) (Figure 1). In eight out of 127 (6.3%), parents/caretakers consulted the geneticist and genetic counseling was performed, but without additional genetic testing (e.g., “no show,” the desire to save material for future use, refrain of genetic testing). In addition to SNP-array (in all cases, *n* = 119), gene panel analysis was used in 22 patients (18.5%), and another 26 patients (21.8%) were additionally tested with Whole Exome Sequencing (WES). Three patients were tested with SNP-array as well as genome panel analysis and WES (2.5%).

In 34 out of 119 patients (28.6%), a causative [possible (*n* = 1, 2.9%), probably (*n* = 4, 11.8%) or certain (*n* = 29, 85.3%)] genetic diagnosis was obtained vs. 85 patients without a causative genetic diagnosis (66.9%) (Table 3). Co-morbidities of epilepsy and autism were only seen in patients without a genetic etiological cause found. In both groups (vs. no genetic etiology), facial dysmorphic features were present in about one out of 3 patients (*n* = 10 in those with genetic etiology vs. *n* = 22 in those without). In 14 (48.3%) children a motor delay was found in the group with a genetic origin for their DLD vs. 23 (28.4%) children in the group without a genetic origin, which difference was not statistically significant [95% CI (29–68%), *p* = 0.052]. In both groups (genetic etiology vs. no genetic etiology) a similar distribution in IQ scores was seen (*n* = 7, 63.6% vs. *n* = 28, 63.6%) with the majority of cases an IQ test result of ≤75 points.

**Table 3:** Comparison in characteristics between children who underwent genetic testing and were diagnosed with a causative genetic diagnosis vs. children without a causative genetic diagnosis.

**Characteristics**	**Patients diagnosed with a causative genetic diagnosis (*n* total = 34) *N*, %**	**Patients without a causative genetic diagnosis (*n* total = 85) *N*, %**	^*^p-value
Epilepsy	0/34 (0)	2/85 (2.4)	0.37
Autism	0/34 (0)	1/85 (1.2)	0.53
Facial dysmorphology	10/32 (31.3)	22/80 (27.5)	0.69
Motor delay^a^	14/29 (48.3)	23/81 (28.4)	0.05
Hearing			
Sufficient	27/34 (79.4)	67/85 (78.8)	0.94
Insufficient	5/34 (17.5)	12/85 (14.1)	0.93
Unclear^c^	2/34 (5.9)	6/85 (7.1)	0.82
IQ			
Intellectual disability	7/11 (63.6)	28/44 (63.6)	1.00
Below average level	4/11 (36.4)	14/44 (33.8)	0.77
Average level	0/11 (0)	2/44 (4.5)	0.47
BSID			
Below average level	11/12 (91.6)	15/17 (88.3)	0.20
Average level	1/12 (8.4)	2/17 (11.8)	0.20
FH of speech and language delay^b^	22/28 (78.6)	47/72 (65.3)	0.20

*n, number*.

a *Delay defined as walking >18 months*.

b *First and second degree family members*.

c *Data from testing inconclusive*.

* *p-value < 0.05*.

Thirty-four children (26.8%) received an underlying genetic diagnosis for their DLD (Table 4). A chromosomal microdeletion or duplication/Copy Number Variation (CNV) was found in 15 (44.1%) patients, gene mutations in 15 (44.1%) patients, repeat expansion in only one patient, and other chromosome abnormalities in 3 patients (8.8 %).

**Table 4:** Genetic outcome of children diagnosed with an etiological cause for developmental language delay (DLD).

**Genetic diagnosis, *n* (34)**	**Possible, *n* (4)**	**Probably, *n* (1)**	**Certain, *n* (29)**	Used techniques^**^
Chromosomal microdeletion or duplication, *n* = 15				
Terminal deletion 2q37.3 (2q37 deletion syndrome, OMIM#600430)	–	–	1	SNP-array
Duplication 3p26.3(CHL1 gene), dup 16p11.2 (16p11.2 dup syn; OMIM#614671)	–	–	1	SNP-array
15q13.2q13.3 deletion (15q13.3 microdeletion syndrome, OMIM#612001)	–	–	2	SNP-array
15q11.2 duplication syndrome^b^ (PubMedID:21359847)	–	–	2	SNP-array
15q11-q13 deletion on maternal allele (Angelman syndrome, OMIM#105830)^*^	–	–	1	SNP-array
16p12.2 (deletion syndrome, OMIM#136570)	–	–	1	SNP-array
16p11.2 deletion (16p11.2 deletion syndrome, OMIM#611913)	–	–	2	SNP-array
16q22.3q24.1 deletion (PubMedID 9182777 and 9409869)	–	–	1	SNP-array
17p12p11.2 deletion (Smith-Magenis syndrome, OMIM#182290)	–	–	1	SNP-array
17q11.2 deletion, overlap with NF1 gene (Neurofibromatosis, OMIM#162200)	–	–	1	SNP-array
22q11 deletion syndrome (DiGeorge syndrome, OMIM#188400)	–	–	1	SNP-array
Duplication 10p15.3 (ZMYND11 gene, OMIM#616083)	1	–	–	SNP-array
Chromosome abnormalities, *n* = 3				
Klinefelter syndrome	–	–	2	Karyotyping
Chromosome 15^a^ (15q11-q13)	–	–	1	Karyotyping
Repeat expansion, *n* = 1				
Fragile-X syndrome, (OMIM#300624; FMR1 gene, >200 repeats)	–	–	1	Repeat expansion test
Gene mutation, *n* = 15				
ANKRD11^c^ (KBG syndrome, OMIM#148050)	–	–	1	WES
DEAF1^c^ (Vulto-van Silfout-de Vries syndrome, OMIM#615828)	–	–	1	WES
MECP2 duplication (OMIM#300005)	–	–	1	SNP-array
*SPTAN1*^c^ (Developmental and epileptic encephalopathy 5, OMIM#613477)	1	–	–	WES
SYN1 (OMIM#300491)	–	–	1	Gene panel
CAMTA1^c^ (Cerebellar ataxia, with developmental delay, OMIM#614756)	–	1	–	WES
*CIC*^c^ (Developmental delay, autosomal dominant 45, OMIM#617600)	1	–	–	WES
FOXP1^c^ (Developmental delay with language impairment OMIM#613670)	–	–	1	WES
BCL11B^c^ (Intellectual developmental dis. with dysmorphic facies, OMIM#618092)	–	–	1	WES
*CLCN4* (X-linked developmental delay, OMIM#300114)	1	–	–	WES
*BRWD3* (X-linked developmental delay syndrome, OMIM#300659)	–	–	1	WES
*KDM5C* (X-linked developmental delay syndrome, Claes-Jensen type, OMIM#300534)	–	–	1	WES
*TRPS1* (Trichorhinophalangeal syndrome, OMIM#190350)	–	–	1	Gene panel
*ARID1B* (Coffin Siris syndrome, OMIM#135900)	–	–	1	SNP-array
*CHD2* (Childhood onset epileptic encephalopathic syndrome EEOC, OMIM #615369)	–	–	1	Gene panel

*n, number; SNP, Single Nucleotide Polymorphism; WES, Whole Exome Sequencin*.

a *paracentric inversion*.

b *No OMIM number available (found in multiple control patients)*.

c *de novo mutation*.

d *Frameshift mutation*.

* *Size of deletion ~4.9 Mb*.

** *Test that confirmed genetic diagnosis*.

## Discussion

### Role of Genetic Analysis in Children With Speech and Language Delays

Considering the fact that a language disorder can be primary or secondary to other conditions and can have a significant impact on the (cognitive) development, a genetic assessment should be considered in a child not meeting the expected developmental milestones. To date, genetic evaluation is usually not part of the primary diagnostic work-up. A causative genetic diagnosis can facilitate counseling of children and parents for possible future difficulties and prognosis. In this retrospective cohort study of children with a newly diagnosed developmental language disorder, a causative genetic origin was found in approximately a quarter of the cases (26.8%). This underlines the potential value of genetic assessment to identify the cause (40).

### Risk Factors for Speech and Language Disorders and the Genetic Origin

A family history of speech and language delay (14–16), dysmorphic features (41) and low intelligence (intellectual disability) (42) are considered to be related with a genetic origin of speech and language delays based on current literature. Therefore, these variables might prompt physicians to consult a geneticist or initiate genetic analyses in such cases. However, in our study, these factors were not significantly related to finding a causative genetic origin for this deficit. This could be attributed to the specific study sample, in the first place because in this study children with DLD were referred to a geneticist for multiple reasons, not only having DLD. Besides this, intelligence level was only tested in half of the children (47.2%) of our cohort, which makes it difficult to generalize these outcomes. By performing intelligence testing more deliberately in children referred for speech and language difficulties, this outcome could provide more insight in the differences between (sub) groups of children with speech and language delays.

### Genetic Analysis in Children With Speech and Language Delays

The median time between initial speech and language assessment and the first genetic consultation was 10 and 12 months between initial assessment and confirmed genetic diagnosis. This time interval indicates that not all cases were directly referred to the geneticist during first speech and language assessment. As stated before, in most practices genetic analysis is not a standard procedure in the diagnostic work-up of these children. At this moment, there are no international standards or guidelines regarding the preferred diagnostic procedures for these children. Moreover, there is a lack of awareness regarding which diagnostic tests are most helpful. Furthermore, a diagnostic delay for a causative genetic diagnosis is very common in children with several types of developmental deficits following presentation of a first symptom (43).

The genetic outcomes of our study were mainly based on SNP array testing: SNP array was performed in 96.6% of the patients, whereas WES was only performed in 19% (in all cases SNP prior to WES). This might be due to the difference in time of implementation of the techniques in clinical practice and the fact that until recently little was known about the diagnostic yield and possibility of unsolicited findings when performing WES in children with language delays. SNP-array techniques are widely available and have been routinely performed in children with a developmental delay since 2008, while WES analyses were, introduced worldwide in 2016. This led to a small number of children tested with this technique in our cohort (42). SNP-array usually detects DNA duplications and deletions (44), whereas gene panel analysis (mutation analyses in selected genes), and WES can detect specific variants/mutations of which the origin can be identified by including the parents in the analysis (45). Nowadays, WES is becoming more and more available and costs are reducing. The 28.6% of cases in which a causative gene was found for the language delay probably constitute an underestimation. A higher yield of causative genes would probably be reached if WES was performed more frequently. However, to date, there is no general guideline available on when and how to perform genetic analysis in children with language delays. This urges the need for development of a standard/guideline for clinical practice in children with this type of disorders.

As stated above, an important aspect to keep in mind is the possibility of unsolicited findings (UF) by using next-generation sequencing technologies, such as WES. Genetic diagnostic tests could cause stress in children/parents and can result in unintended outcomes, such as a new diagnosis, not explaining the specific deficit. Since the, introduction of the WES, research has shown that parents were more likely to accept UF's for medically actionable conditions in childhood (46). For UF's of non-actionable conditions, the preference of the parents was found to not perform genetic analyses or wait until adulthood to preserve the autonomy of the child (46). This indicates the importance of genetic counseling before genetic testing so that such unsolicited findings are addressed in advance. However, the risk of incidental findings is negligible in SNP-array and gene panel testing and only <1% in Whole Exome Sequencing (47). Therefore, considering the potential value to find the cause of DLD in children, genetic analyses needs a serious consideration.

Speech-language pathologists and audiologist are among the first to examine children exhibiting speech and/or language difficulties with or without additional deficits. Recent efforts have been undertaken to increase the knowledge about genetics and genetic testing of these specialists as supported by the American College of Medical Genetics and Joint Committee on Infant Hearing (48). Considering the outcome of our study, we strongly support this initiative to improve the awareness of a potential genetic origin and to counsel patients and their parents for possible further testing.

### Strengths and Limitations

In this study we analyzed the diagnostic outcome of children diagnosed with a developmental language disorder who were referred for genetic analysis. As such, this study provides unique knowledge about genetic outcomes for these children. However, this study has some important limitations. The retrospective nature of the study results in missing data. In addition, the used electronic system did not allow to, include the total number of patients diagnosed with DLD during the selected study period which would have led to a more objective view on the records eligible for inclusion. Secondly, the selection of participants visiting a tertiary center for children with speech and language delays and referred for genetic analysis (in the same center) after DLD diagnosis, is highly specific and limits the possibility to generalize the results. Therefore, these outcomes cannot be extrapolated to the general population. And finally, because a part of the data was based on parental recall and notes of the consulting physician, information bias could occur. However, even with these limitations in mind, the outcome of this study discloses new insights of importance for children with DLD, as it underlines the potential genetic origin of this disorder (or as a part of a wider spectrum). To propose a tiered approach as to which group of patients with DLD will benefit most from an early genetic consult, further studies are needed in children with DLD all receiving a standardized medical and full genetic work up. By doing this, those variables of importance could be selected to guide the diagnostic pathway for each individual patient with DLD. Moreover, this could create insight in whether differences in underlying genetic etiology would result in differences in clinical outcomes of DLD patients.

## Conclusions

In this retrospective study, we assessed the genetic outcomes of children with DLD who were referred to a geneticist. In about a quarter of those children, a genetic origin was confirmed. This study demonstrates the opportunity to identify an underlying genetic etiology in children with DLD. Whether an early genetic diagnosis and knowledge about the genetic cause will lead to better treatment and timely interference is not clear. In order to improve clinical care for patients with DLD, two important research gaps need to be filled before we can make recommendations about genetic testing in children with DLD. First, research should focus on assessing the clinical impact and effect on treatment outcomes of the genetic diagnosis. Secondly, there is a need to develop an approach of stratify children with DLD into specific risk groups so as to determine which of them will benefit from a tier genetic assessment.

## Data Availability Statement

The raw data supporting the conclusions of this article will be made available by the authors, without undue reservation.

## Ethics Statement

The studies involving human participants were reviewed and approved by UMCU Ethics Committee. The patients/participants provided their written informed consent to participate in this study.

## Author Contributions

MP performed retrospective analysis of medical records, scored all demographics, and performed data analysis. VW checked manuscript for adequate nomenclature regarding speech, language delay, and assessed whether corresponding genes could be linked to (known) speech and language problems. HW assisted in describing part of diagnostic work-up regarding speech and language delay (e.g., scoring systems for intelligence/speech and language level). EB assisted in describing genetic part of methods, rewrote genetic diagnoses in fitting jargon (Table 4), performed all OMIM number in Table 4, and checked all genetic diagnoses whether they were explanative for the speech, language delay of the child. IS assisted in writing the result section/ result tables. M-JB evaluated all genetic diagnoses from all the children assessed by a geneticist during the including period whether there were explanative for the speech and language delay of the child/clinical state of the child, and assisted in writing the genetic part of the method. AS assisted in choosing scored variables, method of analysis, and checked every new version of the manuscript. All authors contributed to the article and approved the submitted version.

## Conflict of Interest

The authors declare that the research was conducted in the absence of any commercial or financial relationships that could be construed as a potential conflict of interest.
