# Supplementary material for: Clinical Characteristics and Genetic Etiology of Children With Developmental Language Disorder
Source: Front Pediatr. 2021 Jul 1;9:651995. doi: 10.3389/fped.2021.651995 (PMC8282268; doi:10.3389/fped.2021.651995)
Supplement: Supplementary file 1 [file Table_1.pdf]

**Appendix 1:** Specifications of comorbidities in total cohort, children diagnosed with Developmental Language Delay where genetic analyses was performed.	
	Children with Developmental Language Delay **N= 127, %**
Comorbidities	N= 22
Epilepsy	2 (9.1)
Autism	1 (4.5)
Cleft palate	0 (0)
Squint	1 (4.5)
Cardial	4 (18.2)
Polycystic kidney	0 (0)
Abbreviations: n= number	
