# Supplementary material for: Clinical Characteristics and Genetic Etiology of Children With Developmental Language Disorder
Source: Front Pediatr. 2021 Jul 1;9:651995. doi: 10.3389/fped.2021.651995 (PMC8282268; doi:10.3389/fped.2021.651995)
Supplement: Supplementary file 2 [file Table_2.pdf]

**Appendix 2:** Specifications of difficulties during pregnancy and perinatal emergency in total cohort, children diagnose with Developmental Language Delay (DLD) were genetic analyses was performed.	
	Children with Developmental Language Delay<br>**N= 127, %**
Difficulties during pregnancy*	N= 37
Cardiovascular	7 (18.9)
Respiratory	1 (2.7)
Endocrine	10 (27.0)
Infection	3 (8.1)
Urogenital	1 (2.7)
Pregnancy/childbirth	8 (21.6)
Musculoskeletal	1 (2.7)
Neurologic	1(2.7)
Multiple	5 (13.5)
Perinatal emergency *	N= 11
Cardial	3 (27.3)
Respiratory	5 (45.5)
Cardial and respiratory	3 (27.3)
Abbreviations: n= number	

* Five mothers suffered from pre-eclampsia

Respiratory problems directly after birth scored as perinatal emergency were mostly due to a wrapped umbilical cord (3 patients). Idiopathic respiratory distress syndrome (IRDS) was reported in only one patient. Only one patient suffered from cardiac problems.
