# Supplementary material for: Clinical Characteristics and Genetic Etiology of Children With Developmental Language Disorder
Source: Front Pediatr. 2021 Jul 1;9:651995. doi: 10.3389/fped.2021.651995 (PMC8282268; doi:10.3389/fped.2021.651995)
Supplement: Supplementary file 3 [file Table_3.pdf]

**Appendix 3:** Referrer and reason for referral of children diagnosed with Developmental Language Delay (DLD) to geneticist in Wilhelmina Children's Hospital, Utrecht, the Netherlands	
**Referrer**	**N= 127, %**
Pediatrician	75 (59.1)
Pediatric surgeon	7 (5.5)
Neurologist	19 (15.0)
ENT specialist	20 (15.7)
Psychiatrist	3 (2.4)
Cardiologist	1 (0.8)
**Reason for referral**	**N= 127, %**
Speech and language delay	39 (30.7)
Developmental disorder	44 (34.6)
Dysmorphological features	2 (1.6)
Epilepsy	4 (3.1)
Autism	2 (1.6)
Mental retardation	12 (9.4)
Hearing loss	5 (3.9)
Nasal speech	2 (1.6)
Developmental disorder and dysmorphological features	5 (3.9)
Learning problems	3 (2.4)
Congenital abnormalities	4 (3.1)
Abbreviations: n= number	
