# Supplementary material for: Clinical Characteristics and Genetic Etiology of Children With Developmental Language Disorder
Source: Front Pediatr. 2021 Jul 1;9:651995. doi: 10.3389/fped.2021.651995 (PMC8282268; doi:10.3389/fped.2021.651995)
Supplement: Supplementary file 4 [file Table_4.pdf]

Appendix 4: Performed hearing/language/intelligence tests in children with DLD

Test	Subtest	Domain	Age	Average range
Hearing				
Tonal audiometry		Hearing level	≥4 years	≤ 20 dB PTA (0.5-2 kHz)
VRA	- headphone<br>- free field		7 months -3 years<br>< 7 months	≤ 15 dB<br>≤ 25 dB<br>≤ 30 dB
BOA				*
ABR			≤1 year	≤ 20 dB
Language				
Schlichting test (TBQ)	-	Language perception	2-7 years	85-115
Schlichting test	Vocabulary (WQ)	Vocabulary	2-7 years	85-115
Clinical Evaluation of Language Fundamentals Version IV(CELF-IV)	Receptive language index (RTI)<br><br>Vocabulary (AW)	Language perception<br><br>Vocabulary	5-18 years	85-115
Development/intelligence				
Bayley Scales of Infant and Toddler Development (BSID)		developmental index<br>cognitive scale	1-42 months	90-110
Snijders-Oomen Non-verbal Intelligence test (SON-R)		Non-verbal intelligence	2-7 years	90-110
Wechsler Non-Verbal Scale of Ability (WNV-NL)		Non-verbal intelligence	4-21 years	90-110
Wechsler Intelligence Scale for Children (WISC)		Intelligence<br>general intellectual ability	6-18 years	90 – 109

Abbreviations: PTA; Pure tone average. dB; decibel. VRA; Visual reinforcement audiometry, BOA; behavioural observation audiometry, ABR; Auditory Brainstem evoked Responses.
*limits for hearing levels by BOA testing for sufficient hearing to develop a normal speech and language were according to Williams and Wilkins (23);
